# Gastric Lipoma in a 10‐Year‐Old Patient: A Case Report and Literature Review

**DOI:** 10.1002/pdi3.70055

**Published:** 2026-06-11

**Authors:** Yiwen Huang, Ting Li, Baisha Huang, Peng Li, Xiaochun Zhu, Wuping Ge, Chengwei Chai

**Affiliations:** ^1^ Department of Pediatric Surgery Guangdong Women and Children Hospital Guangzhou Guangdong China; ^2^ Department of Pediatric Surgery Qingxin District Hospital of Women and Children Healthcare Qingyuan Guangdong China

**Keywords:** childhood, gastric lipoma, treatment

## Abstract

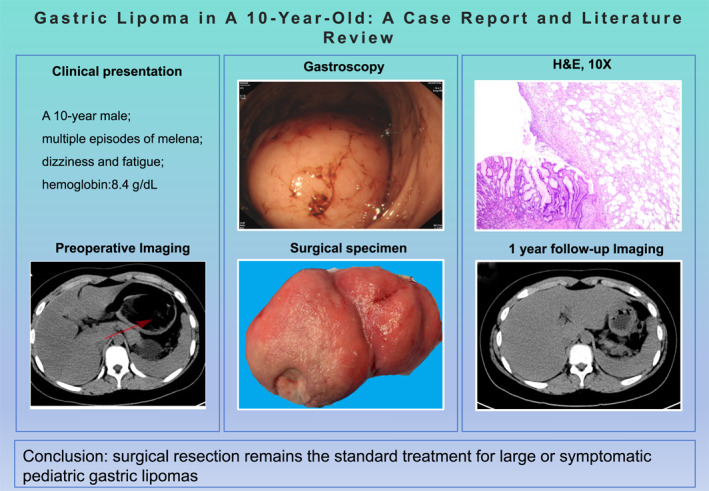

## 1 Background

Gastric lipomas (GLs) are rare in children, accounting for only 1% of gastric tumors [1]; even in adults, they represent only 2% [2]. A total of 6 relevant cases of childhood gastric lipoma have been reported thus far [3, 4, 5, 6]. The tumor is mostly asymptomatic, mainly in the initial stage. However, while their size increases, they may present with symptoms such as abdominal discomfort, dyspepsia, dark stool, and in rare circumstances, upper gastrointestinal (GI) bleeding and gastroduodenal intussusception [4]. We present the case of a male child with GL who presented with multiple episodes of melena, dizziness, and fatigue. We also reviewed some published GL cases to strengthen our understanding of those patients' diagnoses and surgical options.

## 2 Case Description

A 10‐year‐old male presented with multiple episodes of melena with a 10‐day history accompanied by dizziness and fatigue. His parents denied the use of nonsteroidal anti‐inflammatory drugs (NSAIDs) or anticoagulants. Physical examination revealed remarkably pale skin and lips. Clinical laboratory testing revealed an acute drop in hemoglobin (8.4 g/dL) and albumin (36.3 g/L) and an increase in prothrombin time (PT, 13.7 s). Contrast‐Enhanced Computed Tomography (CECT) imaging, shown in Figure 1, revealed a 9.0 × 6.7 cm (maximum cross‐section) fat‐density space‐occupying lesion within the wall of the body of the stomach, suggesting a lipoma. Esophagogastroduodenoscopy revealed a large lipoma, approximately 70 mm in diameter, near the *curvature ventriculi major* and the gastric fundus (Figure 2).

**FIGURE 1:**
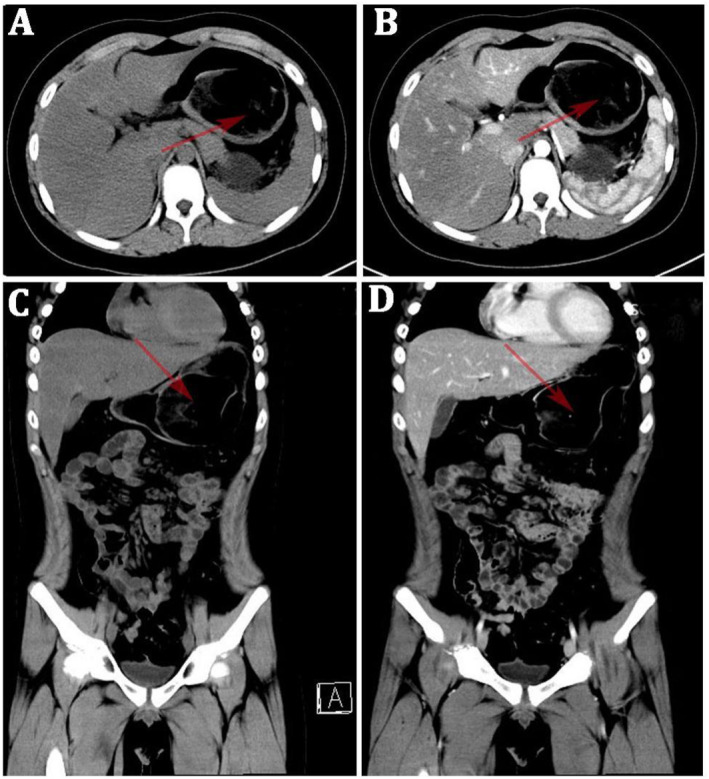
Abdominal computed tomography (CT) revealed a large space‐occupying lesion in the stomach. (A) A CT scan obtained through the body of the stomach shows an axial plane of a low‐density mass (arrow). (B) A contrast‐enhanced computed tomography (CECT) scan obtained through the body of the stomach shows no enhancement in the axial plane of the low‐density mass (arrow). (C) A CT scan obtained through the body of the stomach shows a sagittal plane of a low‐density mass (arrow). (D) A CECT scan obtained through the body of the stomach shows no enhancement over a sagittal plane of a low‐density mass (arrow).

**FIGURE 2:**
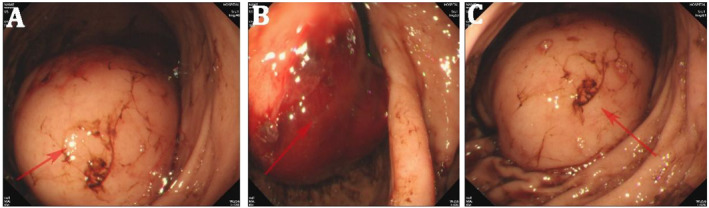
Esophagogastroduodenoscopy revealed a large lesion on the stomach. (A‒C): Endoscopy reveals a mass lesion(arrows) in the body of the stomach, approximately 70mm in diameter.

During the procedure, a 13.0 cm skin incision was made approximately 2.0 cm below the left costal margin to access the gastric tumor. The tumor measured approximately 10.0 × 12.0 cm (the tumor was not totally exposed) and was soft in consistency. A 3.0 × 5.0 cm ulcer was identified on the surface of the gastric mass (Figure 3). No abnormal lymph nodes were identified. The gastric tumor was successfully removed by resecting part of the gastric mucous membrane. The postoperative course went well. Macroscopically, there is a 14.0 × 9.5 × 3.5 cm (Lesion Measurement), partially encapsulated mass with a light‐yellow color and lobulated structures. A description of the gross pathology is as follows: mature and homogenous adipose tissue with focal areas of hemorrhage, localized ulcer, and no evidence of invasion. Immunohistochemistry (S100 [+], CD34 [+], CDK4 [+]) and fluorescence in situ hybridization (FISH) (DDIT3 [−] [7], PLAG1 [−] [8]) confirmed the diagnosis of submucosal GL (shown in Figure 4). At the one‐year post‐operative follow‐up, the patient was doing well with no evidence of symptom recurrence or tumor regrowth on clinical and imaging (computed tomography, CT) evaluation.

**FIGURE 3:**
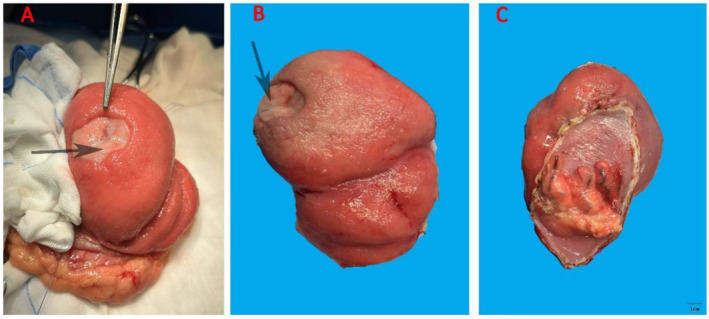
Intraoperative images and an ulcer (arrows) on the tumor. (A) Intraoperative tumor images with an ulcer (arrow). (B) Mucosa view with an ulcer (arrow). (C) The serosal surface of the gastric tumor.

**FIGURE 4:**
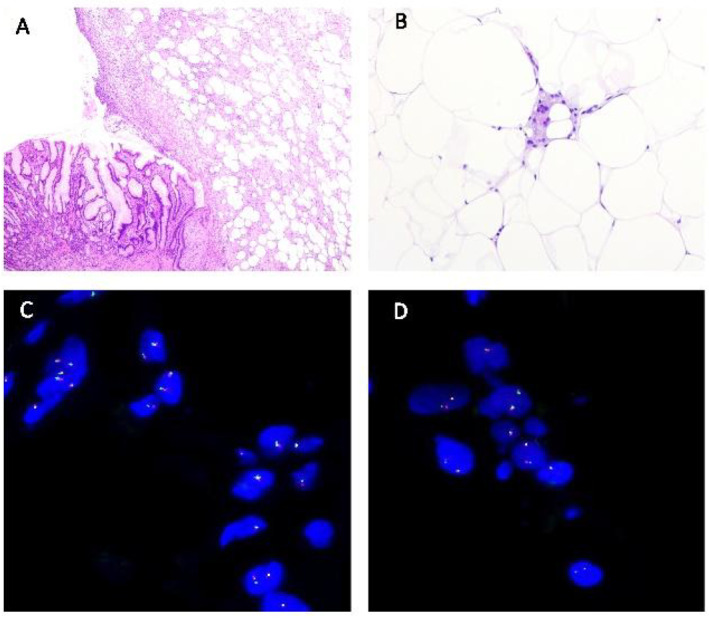
Pathological images of the gastric lipoma. (A) Low‐magnification view (hematoxylin and eosin [H&E], × 10) of the mass, showing a well‐circumscribed lipomatous tumor in the submucosa. (B) High‐magnification view (H&E, × 40) of the mass, highlighting mature adipose cells. (C and D) High‐magnification view (fluorescence in situ hybridization, × 40).

## 3 Discussion

GLs are commonly located in the antrum of the stomach, and 85% of cases arise from adipocytes in the submucosal layer [9, 10]. GLs are rare among stomach tumors, and especially rare in children. Six cases of children have been reported. We summarized the age, sex, tumor size, follow‐up time, and treatment modality of those cases (Table 1). The children were hospitalized due to symptoms that revealed GLs. Frequently, GLs are solitary, and sessile in morphology [13]. Patients with a small stomach lipoma (< 4.0 cm) are usually asymptomatic and can be discovered incidentally [14]. As the tumor gradually grows, symptoms such as gastrointestinal bleeding, epigastric pain, dyspepsia, obstruction, and, rarely, gastroduodenal intussusception can be appreciated clinically [6]. The most common clinical presentation is gastrointestinal bleeding (53%), possibly due to ischemia of the upper mucosa [12, 14]. However, it is common to have more acute presentations with complaints of fatigue, melena, and signs of gastrointestinal obstruction [15]. In our case, the patient's primary symptom was melena for over 10 days. The presentation of GL in the pediatric age group is similar to that in the adult population. To our knowledge, one case of intussusception in children has been reported as being caused by lipoma [6]. The current preoperative diagnosis of lipomas is based primarily on CT [3, 16]: the lesions appearing as well‐circumscribed areas of uniform, fatty density, with a density of −70 to −120 Hounsfield units in the CT examination, a mass that is compressible on fluoroscopy [17], and linear strands of soft tissue attenuation indicating that adipose tissue may be present [18]. Currently, the treatment for GLs is still quite controversial. Alberti et al. [3] reported that an 8‐year‐old girl refused surgery, and after 3 years of follow‐up, she presented no associated symptoms. With the development of endoscopic technology, some surgical doctors have attempted endoscopic resection. Yu et al. [19] reported a series of 15 adult patients with lipoma; all lipomas were successfully removed endoscopically. It is worth noting that in their cases, GLs with a stalk can be resected entirely endoscopically, except for large GLs, which need multiple resections. Most lipomas (4.2 × 8.0 cm) were removed by coagulation current, but a residual tumor was left on the mucosa. One endoscopic resection has been reported thus far in children. Beck et al. [12] reported a 13‐year‐old male with several bouts of hematemesis. Endoscopy found a 3.0 × 4.0 × 8.0 cm mass, with a smooth surface and basal ulceration, growing on the anterolateral wall of the antrum. Endoscopic polypectomy was immediately scheduled and attempted; however, they soon stopped the surgery because of severe pain whereas the tumor remained firmly fixed. Finally, laparotomy under general anesthesia was scheduled for removal of the lesion.

**TABLE 1:** Clinical data of 7 patients (including the one presented in this article) with lipoma of the stomach reported in the published literature.

Serial number	First author	Publication year	Cases	Age (years)	Sex	Tumor size	Physiology	Surgical procedures	Follow‐up time
1	Stefan	1966 [11]	1	12	Female	—	—	—	—
2	McCombe	1988 [6]	1	12	Male	—	Lipoma	Laparotomy	6 months
3	Alberti	1999 [3]	1	11	Female	—	—	Laparotomy	3 years
4	Beck	1997 [12]	1	13	Male	3.0 × 4.0 × 8.0 cm	Lipoma	Laparotomy	—
5	Antoniou	2007 [4]	1	10	Male	4.0 × 3.5 cm	Fibrolipoma	Laparotomy	2 years
6	Zamee	2010 [5]	1	12	Male	12.0 × 8.0 cm	Lipoma	Laparotomy	1 year
7	Huang	2026	1	10	Male	14.0 × 9.5 × 3.5 cm	Lipoma	Laparotomy	1 year

*Note:* —, not reported in representative literature.

Whether to treat asymptomatic GLs is currently controversial. Antoniou et al. [4] reported that a 10‐year‐old male complained of merely vague epigastric pain. Gastroscopy revealed a smooth, broad‐based, rounded polypoid mass measuring approximately 4.0 cm with no surface ulceration. They decided to perform no treatment and suggested an intense observation period. Next, the child was readmitted with severe hematemesis and melena and then underwent surgical excision. McCombe and Orr [6] reported that a 2‐year‐old male presented with forceful vomiting that was pale after meals and was resuscitated and subsequently investigated. When following these investigations, his vomiting recurred, urgent Laparotomy was performed, and gastroduodenal intussusception, which caused the above symptoms, was identified. Zameer et al. [5] reported that a 12‐year‐old male presented with active hematemesis and malena for 6 months and was treated with multiple blood transfusions. After a series of examinations, including ultrasound, contrast‐enhanced computed tomography (CECT), and endoscopy, the child underwent laparotomy. Stefan et al. [11] reported that a 12‐year‐old female presented with a massive life‐threatening hemorrhage. The reference, the radiological investigations and the endoscopic findings complement each other in diagnosing GLs. Finally, determining the diagnosis relies on histology. The treatment of lipoma depends on its symptoms. Although endoscopy [20], laparoscopy and laparotomy are currently available in adults, small lesions (< 2.0 cm) can be removed endoscopically by submucosal dissection, snare polypectomy, band ligation, or unroofing [21, 22]. In adults, laparoscopic resection is advised for lipomas less than 6.0 cm in diameter [23]. Termos et al. [24] reviewed larger GLs over 8.0 cm in adult patients who underwent Laparotomy. In contrast to adult cases, pediatric GLs often present with acute symptoms or large tumor size at diagnosis, making laparotomy the management of choice. For asymptomatic children, however, conservative management with active surveillance may be considered.

## 4 Conclusion

GLs are typically asymptomatic but may cause nonspecific symptoms when present. Diagnosis can be established by CT scan and confirmed with endoscopy and biopsy. How to determine the optimal treatment strategy is a controversial question, especially in children, who are often admitted and under inspection for urgent symptoms or large GLs. Consequently, open laparotomy has traditionally been the common approach. The application of laparoscopic or endoscopy‐assisted techniques in pediatric GL surgery holds promise for achieving superior therapeutic outcomes.

## Author Contributions

Chengwei Chai offered the funding. Yiwen Huang and Ting Li wrote this issue and collected these materials. Baisha Huang, Peng Li, and Wuping Ge all participated in this operation. Xiaochun Zhu checked this issue's reading.

## Funding

This study was supported by the Science and Technology Project of Guangzhou (Grant No. 1.202201020594) and Guangdong Provincial Department of Science and Technology (Grant No. 2023A1414020012).

## Ethics Statement

The issue involved in this project has been approved by the ethics committee of Guangdong Women and Children Hospital. (Ethics approval number: 20251012). We obtained informed consent from the parents and the child for publication.

## Conflicts of Interest

The authors declare no conflicts of interest.

## Data Availability

The original source material of the case may be made available at any time (mainly from Chengwei Chai or Yiwen Huang), and it is determined that the data may be cited and published.
